# Multiple Methods Synergistically Promote the Synchronization of Somatic Embryogenesis Through Suspension Culture in the New Hybrid Between *Pinus elliottii* and *Pinus caribaea*

**DOI:** 10.3389/fpls.2022.857972

**Published:** 2022-04-25

**Authors:** Fengqing Li, Jiabao Yao, Lingfeng Hu, Jinhui Chen, Jisen Shi

**Affiliations:** ^1^Key Laboratory of Forest Genetics and Biotechnology of Ministry of Education of China, Co-Innovation Center for Sustainable Forestry in Southern China, Nanjing Forestry University, Nanjing, China; ^2^Experimental Center of Subtropical Forestry, Chinese Academy of Forestry, Fenyi, China

**Keywords:** *Pinus elliottii* × *Pinus caribaea*, embryonic calli, somatic embryogenesis, synchronization, suspension culture

## Abstract

Pinus elliottii × *Pinus caribaea* is an interspecific pine hybrid of major economic importance. Somatic embryogenesis and plant regeneration in *P. elliottii* × *P. caribaea* on solid medium have been reported previously; however, a current limitation is the lack of a stable and effective method for its commercial use. The objective of this study was to establish a suspension culture system and evaluate the effect of multiple methods synergistically on the synchronization of embryo development in *P. elliottii* × *P. caribaea*. For the former, a protocol to initiate and establish a suspension culture system of *P. elliottii* × *P. caribaea* was presented. Based on biomass growth, the growth of embryogenic calli (EC) followed an S-shape curve in suspensions grown for a 15-day period, and the exponential phase of cell suspensions was reached between days 3 and 6. The initial packed cell volume (PCV) and revolutions per minute (rpm) have a significant effect on the proliferation of EC, and the highest proliferation multiple reached 6.86 (±0.06) at the initial density of 5 ml PCV under a 9–10 days transfer interval in the dark on a rotary shaker at 70 rpm. For the latter, the influence of abscisic acid (ABA), ammonium (NH_4_^+^), nitrate (NO_3_^–^), low temperature, and polyethylene glycol (PEG) on somatic embryogenesis was very significant. When EC were suspended in the medium at a presence of 37.84 μM/L ABA, as many as 274 mature cotyledonary embryos/ml PCV of cells were thereafter formed in the mature medium, and 266 somatic embryos were obtained on mature medium after suspension culture in liquid medium containing 10 mmol/L NH_4_^+^ and 30 mmol/L NO_3_^–^. Furthermore, reducing the concentration of 2,4-dichlorophenoxyacetic acid gradually and at 4°C incubation for 12 h in the initial exponential phase could promote the synchronization of somatic embryogenesis, which resulted in 260 mature cotyledonary embryos. This suspension culture system and method of synchronic control can be used in the large-scale production of *P. elliottii* × *P. caribaea* seedlings.

## Introduction

The new interspecific hybridization between *Pinus elliottii* var. *elliottii* and *Pinus caribaea* var. *hondurensis* was successfully bred by scientists at Queensland Forestry Research Institute in Australia in 1955. The hybrid possessed an outstanding growth advantage (Mutete et al., 2015; Zhao et al., 2018); it was called “Shi-Jia-Song” in China and was introduced into China in the late 1970s, with a strong potential for fast-growing plantation establishment in southern China (Zhao et al., 2006). Due to its highly improved characteristics, industrial demand for this hybrid is increasing, and the traditional seed propagation (seed orchard) is struggling to meet the requirements of large-scale mass production in a short period of time.

Somatic embryogenesis (SE) is a useful system for the mass propagation of plant material *in vitro* (Gupta and Timmis, 2005; Kaur and Kapoor, 2016; Zhong et al., 2017) and is a morphogenetic process by which somatic cells go through a series of morphological and biochemical changes that result in the formation of a somatic or non-zygotic embryo capable of regenerating plants (Aguilar-Hernández and Loyola-Vargas, 2018; Garcia et al., 2019). Therefore, SE has become a good system for theoretical studies in plant development, especially the molecular processes of embryo development, genetic transformation, endangered species conservation, and artificial seed production (Quiroz-Figueroa et al., 2006; Aquea and Arce-Johnson, 2008; Hernández et al., 2011; Morel et al., 2014; Kaur and Kapoor, 2016; Ahn et al., 2019). However, SE usually occurs at low frequency and poor synchronization, that is to say, embryos at different developmental stages are always present in a given culture. Therefore, attempts have been made to establish a system in which embryogenesis occurs synchronously and at high frequency.

An efficient, synchronous SE system is of particular importance for commercial micropropagation and for synthetic seeds production and physiological *in vitro* studies (Al-Khayri and Al-Bahrany, 2012). Such system has recently been established in suspension culture in many species, i.e., carrots (Osuga and Komamine, 1994), sweet potato (Torres et al., 2001), date palm (*Phoenix dactylifera*) (Al-Khayri and Al-Bahrany, 2012), and Douglas-fir (*Pseudotsuga menziesii*) (Gupta and Timmis, 2005). Cultures grown in the liquid medium have shown characteristics of well dispersion, rapid growth, and good repeatability, and it is easier to realize the synchronous generation of somatic embryos than that on the solid medium (Song et al., 2018). However, SE is a complex developmental process, and the synchronous development of somatic embryos is affected by exogenous plant growth regulators (PGRs), osmotic pressure, temperature, and so on, which trigger a serious transformation of the morphology of the cell and biochemical, genetic, and epigenetic changes (Aguilar-Hernández and Loyola-Vargas, 2018). Cells fractionation by sieving and density gradient centrifugation in Ficoll solution is considered as a suitable method for the separation of subpopulations with different embryo development stages in some angiosperm species (Osuga and Komamine, 1994; Tsolova et al., 2007). However, compared with the somatic embryo of angiosperms, somatic embryo for the most gymnosperm initiated from immature zygotic embryos was considered as a result of a continuous process of cleavage polyembryony in zygotic embryo, that is, embryogenic suspensor mass (ESM) was composed of an embryonic head and highly vacuolized and extended cells. Therefore, chemical methods, such as regulating nitrogen source or relative proportion of oxidized versus reduced nitrogen (Zayed et al., 2012; Pìnèík et al., 2015; Wang et al., 2019), and low temperature should be adopted to obtain synchronization of SE. Synchronization of SE has been achieved by low temperature in several species, such as *Taxus chinensis* (Mei et al., 2001) and *Prunus avium* (Reidiboym-Talleux et al., 2000). The role of nitrogen forms on the synchronization of SE was also reported in carrot (*Daucus carota* L.) (Mashayekhi-Nezamabadi, 2000) and tomato (*Lycopersicon esculentum* Mill.) (Shomali et al., 2018).

In conifers, descriptions of SE were first described by Hakman et al. (1985) in *Picea abies* and by Nagmani and Bonga (1985) in *Larix decidua*. To date, six families and 14 genera of conifers have made great progress in clonal somatic embryogenesis according to incomplete statistics (Klimaszewska et al., 1997). Recently, the potential of SE as a tool for rapid clonal propagation of conifers has been emphasized in China (Hu et al., 2019; Sun et al., 2019; Liu et al., 2020). SE and plant regeneration using immature zygotic embryos of hybrids between *P. elliottii* and *P. caribaea* on the solid medium have been reported (Nunes et al., 2018; Hu et al., 2019). Nevertheless, compared to SE on solid medium, SE in liquid medium tend to proliferate much faster and possess higher efficiency, and this technique has been used in the micropropagation of other conifer trees (e.g., *P. menziesii* and *Pinus taeda*) (Klimaszewska et al., 1997; Gupta and Timmis, 2005), yet such a system had not been reported in *P. elliottii × P. caribaea*. To establish and optimize the suspension culture system of *P. elliottii × P. caribaea*, the main factors (initial PCV and rpm) influencing the proliferation of EC were compared in this study; on this basis, the synchronization of SE was explored by adjusting abscisic acid (ABA), nitrogen forms, temperature, and polyethylene glycol (PEG), aiming to achieve rapid proliferation of ECs and synchronous SE with steady high conversion rate and regeneration capacity and to provide a preliminary technical basis for establishing a large-scale culture system by bioreactors.

## Materials and Methods

### Plant Material and Culture Conditions

**FIGURE 1:**
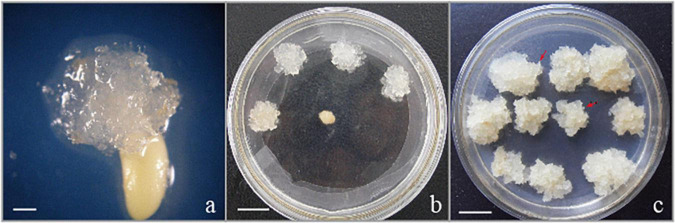
Initiation and proliferation of somatic callus in the hybrid *P. elliottii* × *P. caribaea*. **(a)** Cell proliferation initiated at the micropylar end of the megagametophyte, which was the same as Liao and Amerson (1995) described. **(b)** Embryogenic callus subcultured on solid medium to proliferate. **(c)** White, transparent callus, rapid proliferation. The obvious structure of embryogenic suspensor mass composed of embryo head and highly vacuolated and elongated cells is visible under the microscope (red arrowheads). Scale bar = 1 cm.

The EC used in the present research were induced from immature zygotic embryos (cleavage poly-embryo or dominant embryo stage) (Figure 1a) from control-pollinated cones that had been collected in Hongling seed orchard, Guangdong, China. The EC subcultured periodically on solid medium with the transfer interval of 2 weeks were characterized as white or light brown in color, translucent or semi-translucent (Figures 1b,c), and embryogenic suspensor mass (ESM) visible under a stereo microscope (red arrowheads in Figure 1c). This proliferated EC (Figure 1c) were selected for suspension culture in the liquid medium. EC (approximately 2.5 g) subcultured on solid medium for 2 weeks were transferred to 250 ml flasks that contained 50 ml of proliferation medium for the establishment of suspension culture. The liquid proliferation medium contained the same PGRs and additives as the solid medium. The dishes or flasks containing EC were incubated in dark conditions, at 23 ± 1°C.

### The Composition of Culture Medium

#### Induction of Embryogenic Calli

The initiation medium was composed of DCR basic medium (Gupta and Durzan, 1985) containing basal salts and vitamins, supplemented with 9.05 μM/L 2,4-dichlorophenoxyacetic acid (2,4-D), 4.44 μM/L 6-benzyladenine (6-BA), 2.32 μM/L kinetin (KT) (three of them from Dingguo Changsheng Biotechnology Co., Ltd., China), 3.0% (w/v) maltose, 450 mg/L glutamine, 500 mg/L casein hydrolyzate (CH), 200 mg/L inositol, and 1.8 g/L gellan gum (additives and Gelrite from Sigma-Aldrich Co., St. Louis, MO, United States). The PGRs contained induction medium was a modified Shi’s (2005).

#### Maintenance and Proliferation of Embryogenic Calli

To maintain the ability and obtain the rapid proliferation of SE, the EC grown on solid-modified P6 medium (Pullman and Johnson, 2002; Shi et al., 2008) supplemented with 4.52 μM/L 2,4-D, 2.22 μM/L 6-BA, 2.32 μM/L KT, 3.0% (w/v) maltose, 1,000 mg/L glutamine, 500 mg/L CH, 1,000 mg/L inositol, and 1.8 g/L Gelrite (Sigma-Aldrich Co., St. Louis, MO, United States). The pH of the medium was adjusted to 5.8 ± 0.1 with NaOH or HCl before autoclaving at 121°C at 1.10 × 10^5^ N/m^2^ for 17 min (autoclave SMS ASVE, Guangzhou, China). To preserve the SE ability of EC over the long-term culture, EC should be subcultured alternately on solid (stationary) and liquid medium (rotated at 70 rpm), with the transfer period of 14–20 days on solid medium and 7 days in liquid medium, both of them subcultured in the dark at 23 ± 1°C.

#### Maturation Medium of Somatic Embryos

The maturation medium consisted of P6 basic medium supplemented with 48.5 μM/L abscisic acid (ABA), 14.5 μM/L gibberellin (GA) (both from Sigma-Aldrich Co., St. Louis, MO, United States), 60–120 g/L PEG 8000, 1,000 mg/L myoinositol, 500 mg/L CH, 500 mg/L glutamine, 3% (w/v) maltose, 2.0 g/L activated carbon, and 6.5 g/L gellan gum (Dingguo Changsheng Biotechnology Co., Ltd., China), pH = 5.8. For each maturation treatment, at least three replicates were cultured for 6–7 weeks in the dark, at 23 ± 1°C.

### The Establishment and Optimization of a Suitable Suspension Culture System

The EC (approximately 2.5 g), subcultured 6–7 weeks periodically and grown for 2 weeks on solid medium, were transferred to 250 ml flasks containing liquid medium that supplemented the same additives as the solid medium, according to a mass: volume ratio (m/v) of 5%. The flaks with suspension cultures were sealed with a double layer of breathable sealing film and kept on gyratory shaker at 70–110 revolutions per minute (rpm) and 23 ± 1°C in the dark. In the first subculture, the intact EC were separated from the well-dispersed suspension cells to synchronize the stage of the culture. After the establishment of cell suspensions, the medium was replaced with the fresh one on a weekly basis. At the end of one subculture period, the cell suspensions were transferred to a 100 ml measuring cylinder to obtain the packed cell volume (PCV) of cells. PCV was measured according to the method described by Gupta and Timmis (2005). A certain PCV was pipetted into a 250-ml flask containing fresh liquid medium for proliferation experiments.

To determine the effect of the initial cell density, PCV of different volumes of EC (3.0, 4.0, 5.0, 6.0, and 7.0 ml) were added to 250 ml flasks and the fresh medium was added to a final volume of 50 ml to the establishment of cell suspensions. These flasks were rotated at 70 rpm and 23 ± 1°C in the dark. The proliferation coefficients were recorded after 3 × 7 days cycles and calculated as follows: Proliferation multiple = PCV after 7 days of culture/the initial PCV of inoculated ECs.

The effect of rpm (70, 90, or 110) was also studied with initial cell density (5 ml PCV). The transfer interval was 7 days and 20 days in the last culture period. PCV was measured as described above, and the pH of the medium and the weight (fresh and dry) of ECs were recorded every 4 days during a 20-day continuous culture period.

### Synchronization Control of Somatic Embryogenesis in Liquid Culture

Well-scattered cells of EC, composed of embryonal cells and suspensors, were called the embryogenic suspensor mass (ESM). The suspension ESM were cultured in the liquid proliferation medium of P6 supplemented with the same additives and PGRs as on the solid medium to maintain proliferative growth. Then, ESM was subcultured in the fresh medium at a ratio of 1:9 (v/v) by transferring 5 ml PCV to a 250-ml flask containing 45 ml of fresh liquid medium. The flask was rotated at 70 rpm at 23 ± 1°C in the dark and with a transfer interval of 9 days. To induce the synchronization of SE, different concentrations of ABA, nitrogen sources, and combinations of 2,4-D and low-temperature treatment were tested.

#### Effect of Abscisic Acid Concentration on Somatic Embryogenesis

To test the effect of ABA concentration (18.92, 37.84, and 75.68 μM/L) on the synchronization of SE in suspension culture, ABA was added to the liquid medium devoid of plant growth regulators (PGRs).

#### Effect of Nitrogen Forms and Concentrations

To test the effect of forms and the proportion of nitrate and ammonium on the synchronization of SE in suspension culture, a two-factor experimental design was used with four repetitions. Suspension cultures were inoculated in a P6 medium already modified with different nitrogen forms. Three concentrations of NH_4_^+^ (5, 10, and 20 mM/L) and NO_3_^–^ (10, 20, and 30 mM/L) were tested. In the experiment, care should be taken to ensure that the sum of positive ions equals the sum of the negative ions. Therefore, K^+^ and Cl^–^ were used to balance ions. Nitrate and ammonium salts are produced in Guangzhou chemical reagent factory, China.

#### Combination of 2,4-D and Low-Temperature Treatments

During subculture in the liquid medium, the PGRs were gradually reduced until 0. At each subculture, 2,4-D, BA, and KT decreased by 1.81, 0.44, and 0.46 μM/L, respectively. At the same time, under continuous culture for 9 days, the suspension ESM after culturing for 4 days was placed at 4, 10, and 23°C for 12 h, and then, it was recultured at 23 ± 1°C in the dark. During each periodic culturing, revolutions per minute remained the same.

After treatments of synchronization, 1 ml PCV of ESM was plated on a filter paper, and then, the sterile filter paper (9.0 cm in diameter) was placed on the surface of the maturation medium. For each maturation treatment, at least three replicates were incubated for 6–7 weeks in the dark, at 23 ± 1°C.

### Effect of Polyethylene Glycol Concentration on Somatic Embryogenesis in the Mature Culture Stage

To test the effect of PEG concentration (60, 80, 100, and 120 mg/L) on somatic embryo maturation, PEG was added to the mature medium. After periodic culturing, the suspension cells were established, and then, they were inoculated in the medium without any PGRs for the last culture period. The suspension ESM with 1 ml PCV was transferred to a mature medium with different concentrations of PEG.

### Germination and Plant Conversion

Mature cotyledonary embryos were transferred to PGR-free solid DCR basal medium (Gupta and Durzan, 1985) with activated carbon (AC) for germination. Culture plates were incubated for the first 3–7 days in the dark at low temperature (4°C). This was to allow the growth of the radicular ends. The plates containing rootless cultures were then transferred to light at 25 ± 2°C. One month after germination, somatic embryos were transferred to rooting medium containing 1.07 μM/L α-Naphthylacetic acid (NAA), 2.46 μM/L indole-3-butyric acid (IBA), and 2.5 g/L AC. The somatic embryo plantlets that transformed successfully were subcultured 2–3 times to obtain complete plantlets (at a height of 3–4 cm, with roots that were 2–3 cm long).

### Data Calculation and Statistical Analysis

Proliferation multiple was calculated using the PCV method to determine the growth trend of each treatment. Measurement of PCV was done during every transfer period using a cylinder marked with a precise scale. Fresh biomass was measured in the final culture after the medium was filtered and washed with deionized water by vacuum filtration. The number of somatic embryos formed from each treatment (per dish contained 1 ml PCV of ESM) was recorded after 6–7 weeks of culture on the mature medium. Normal, mature cotyledonary embryos were characterized by plump cotyledons, elongated hypocotyls, and milky white or light yellow color. The statistical method of mature cotyledonary embryos was based on the characteristic parameters proposed by Le et al. (2021). The developmental stage of somatic embryos was screened under a stereomicroscope (Leica MZ16, Wetzlar, Germany).

Data were analyzed by a one-way ANOVA and a two-way ANOVA, and when necessary, data were transformed to achieve normality and equality of variance. A Tukey’s multiple comparison test was performed at the probability level of 0.01 using SPSS version 18.0 (IBM, United States). Data are presented as mean ± standard error.

## Results

### Establishment of the Embryogenic Cell Suspensions

**FIGURE 2:**
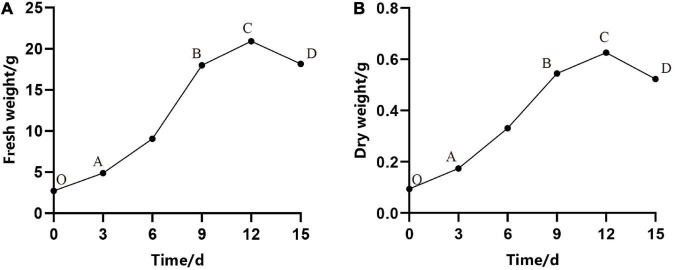
Growth curves of embryogenic calli (EC) of *Pinus elliottii* × *Pinus caribaea*. **(A)** The change of total fresh weight over time. **(B)** The dry weight with time. O–A, A–B, B–C, and C–D represent that the cell was in lag phase, exponential phase, stationary phase, and aging phase, respectively.

In our experiment, we observed that the proliferation rate (i.e., the increase in fresh weight and dry weight over time) of ESM followed an S-shape curve. As shown in Figure 2, under continuous culture for 15 days, the curve went through four stages: lag phase (O–A), exponential phase (A–B), stationary phase (B–C), and aging phase (C–D) (Figures 2A,B). Owing to the damage caused by cutting, burning, and so on, it took several days to recover the normal growth of ESM, which may be due to the adaptation and establishment of colonization of ESM in the fresh medium. It can be seen from Figure 2 that in the first 3 days, the cells were in the lag phase, and then, they entered the exponential phase. At this stage, the fresh weight of the cells increased rapidly, and the dry matter also accumulated rapidly, that is, the cell growth and material accumulation showed a high degree of synchronization. As time in culture increased, the EC eventually turned brown and became necrotic at the stationary and decline stage; meanwhile, the biomass of cells decreased rapidly. Therefore, we concluded that the optimal transfer period was 9–10 days of *P. elliottii* × *P. caribaea*.

**FIGURE 3:**
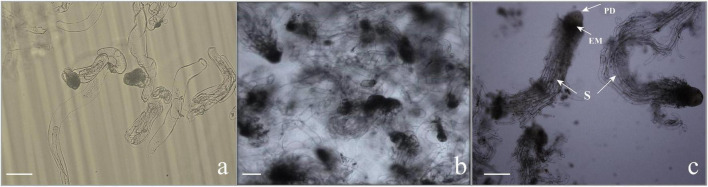
Microscopic characteristics of somatic embryogenesis. **(a)** Callus in the proliferation stage. The embryonic head of the filamentous embryos was composed of small, dense cytoplasmic cells, and the suspensor apparatus was formed by elongated cells with large vacuoles. Scale bar = 100 μm. **(b,c)** Morphology of embryogenic cells growing in the liquid medium subjected to synchronization. **(b)** Embryos stained with toluidine blue, consisting of the embryo proper and embryo suspensor. Scale bar = 200 μm. **(c)** The early somatic embryo consists of a single embryo with a globular compact embryonal mass with a protoderm-like layer and suspensor-like cells (S). Scale bar = 200 μm.

Subculturing every 9–10 days in the liquid medium can not only realize efficient proliferation of EC but also maintain the ability of somatic embryogenesis for a long time, that is to say, a stable suspension cell line was necessary for the efficient proliferation of EC and somatic embryogenesis. Microscopic observation showed that the ESM obtained by suspension culture was loose, and the suspensor apparatus was formed of elongated cells with large vacuoles (Figure 3a).

### Embryogenic Calli Proliferation in Liquid Culture

Our experiment showed that with increasing of initial cell density, the proliferation multiple of ECs increased and the maximum appeared at the initial density of 5 ml PCV, then the proliferation multiple decreased (Table 1). The highest PCV and proliferation multiple reached 34.3 (±0.31) ml and 6.86 (±0.06), respectively. In addition, the phenomenon of clumps (a large number of cell clusters gathered together) appeared on the fifth day after subculture with a continuous increasing initial density of PCV. After centrifugation of cell suspension at 120 × *g* for 10 min, the ESM agglomerated and floated on the top of the liquid surface, resulting in a larger PCV than other treatments; nevertheless, the proliferation multiple decreased significantly. Furthermore, the liquid medium became turbid and the EC eventually turned brown. The ANOVA showed that the initial density had a significant effect on PCV and proliferation multiple of EC (*P* < 0.01).

**TABLE 1:** Effects of the initial density on the dense cell volume and proliferation multiple of *Pinus elliottii* × *Pinus caribaea* embryogenic calli (EC).

Items	Initial dense cell volume/ml				
	3	4	5	6	7
Dense cell volume/ml	19.6 ± 0.17e	27.4 ± 0.39d	34.3 ± 0.31a	35.0 ± 0.23c	36.8 ± 0.16b
Proliferation multiple	6.54 ± 0.06b	6.85 ± 0.10a	6.86 ± 0.06a	5.83 ± 0.04c	5.26 ± 0.02d

*The different lowercase letters in the same column indicate a significant difference (p < 0.01) between treatments based on Duncan’s test.*

**FIGURE 4:**
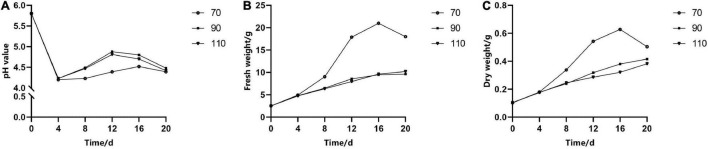
The effect of revolutions per minute (rpm) on the change curves of somatic embryogenic calli (EC) in *Pinus elliottii* × *Pinus caribaea* in pH **(A)**, fresh weight **(B)**, and dry weight **(C)** over time.

The pH of liquid medium over time had the same change trend under different rpm, but the fresh weight and dry weight varied significantly. In the first 4 days of a culture period, the pH of the medium decreased rapidly from 5.8 to 4.5 (Figure 4A); meanwhile, cell growth was relatively slow. When rotated at 70 rpm, the fresh weight and dry weight increased exponentially in the following period (4–16 days) (Figures 4B,C), but the medium pH changed slightly. However, the pH of the medium rotated at 90 and 110 rpm increased more quickly than that of the medium rotated at 70 rpm, while the proliferation rate (i.e., the increase in fresh weight and dry weight over time) increased slowly. It could also be seen that the fresh weight and dry weight had a similar change trend during the subculture stage.

### Synchronization Control and Morphological Observation of Somatic Embryogenesis

#### Effect of Abscisic Acid on Synchronous Somatic Embryogenesis

**FIGURE 5:**
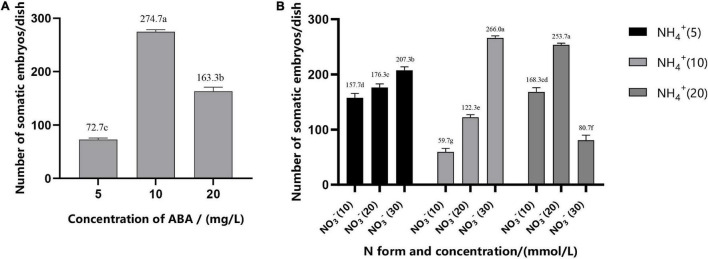
Effect of ABA concentration **(A)**, N form, and concentration **(B)** on the synchronization of mature cotyledonary embryos in *Pinus elliottii* × *Pinus caribaea*. The different lowercase letters indicate significant differences (*P* < 0.01) between treatments based on Duncan’s test. Data represent the mean ± SD of three replicates.

Addition of ABA during suspension culture can improve the synchronous somatic embryogenesis according to the experimental results. After periodic incubation on ABA-containing medium but not on other PGRs, the suspension cultures were transferred to the mature culture medium. The number of mature somatic embryos was estimated after 6–7 weeks of growth. The results showed that concentrations of ABA in the medium in the suspension culture stage had a strong impact on the synchronous maturation of somatic embryos, reaching a highly significant level (Table 2); with the increase of ABA concentration, the highest number of mature cotyledonary embryos were achieved at 37.83 μM/L, with the average number of mature cotyledonary embryos of 274.7, which were induced from 1 ml PCV of cell suspension (Figure 5A).

**TABLE 2:** Variance analysis of the effects of different ABA and PEG concentrations, N source, and temperature on synchronous somatic embryogenesis of *Pinus elliottii* × *Pinus caribaea.*

Factors	Degrees of freedom (df)	Sum of squares (SS)	Mean square (MS)	*F* value	*P*
ABA	2	61510.22	30755.111	67.102	0.009**
NH_4_^+^	2	12583.444	6291.722	18.358	0.000**
NO_3_^–^	2	2923.111	1461.556	1436.623	0.056
NH_4_^+^× NO_3_^–^	4	64445.222	16111.306	4.265	0.002**
T (°C)	2	39096.243	19548.102	37.787	0.000**
PEG	3	12533.521	4177.833	6.966	0.006**

The mean difference is significant at the 1% level. **Indicates significant level at P < 0.01.

#### Effect of N Form and Concentration on the Synchronization of Somatic Embryos

The variance analysis results showed that the concentration of NH_4_^+^ and the interaction between NH_4_^+^ and NO_3_^–^ had an extremely significant effect on SE, but the effect of the concentration of NO_3_^–^ was not significant (Table 2). The number of mature cotyledonary embryos induced under NH_4_^+^ concentration at 5 and 10 mM/L increased with the increase of the nitrate (NO_3_^–^) concentration. When the total nitrogen concentration was 40 mM/L (10 mM/L NH_4_^+^ and 30 mM/L NO_3_^–,^ or both of them 20 ml/L), the number of somatic embryos reached the maximum; the average was 266.0 (±4) and 253.7 (±3), respectively (Figure 5B).

#### Effect of the Combination of 2,4-D and Temperature Treatments on the Synchronization of Somatic Embryos

**FIGURE 6:**
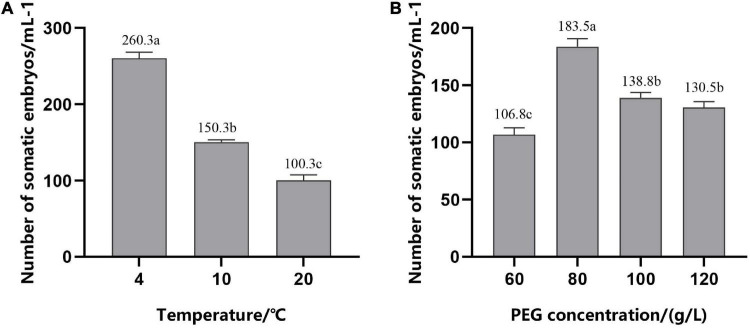
Number of mature cotyledonary somatic embryos per ml of packed cell volume (PCV) obtained from solid maturation medium after culturing for 6–7 weeks. **(A)** In suspension culture, embryogenic suspensor mass (ESM) was treated at different temperatures for 12 h in the period of exponential growth of the culture cycle. **(B)** Cells were cultured on suspension medium without PGRs and then transferred to solid maturation medium with different concentrations of PEG, 45.2 μM/L ABA and 14.5 μM/L GA. The different lowercase letters indicate significant differences (*P* < 0.01) between treatments based on Duncan’s test. Data represent the mean ± SD of three replicates.

To improve the synchronization of somatic embryogenesis in hybrid pine, EC were inoculated in the medium with gradually reduced 2,4-D (0–1.52 μM/L) and cultured at different temperatures for 12 h on the forth day after refreshing the medium. We found that, after 6–7 weeks of culture on the mature medium, low temperature (4°C) for 12 h during suspension culture enhanced somatic embryogenesis efficiency, with average numbers of 260.3 (±8) mature cotyledonary embryos per ml PCV of ESM, while the average number of embryos was 150.3 (±3) at 10°C and 100.3 (±7) at 20°C, respectively (Figure 6A). Temperature had a significant effect on the synchronization of somatic embryogenesis, i.e., at the stage of exponential proliferation, low temperature (4°C) was beneficial to somatic embryogenesis.

### Effect of Polyethylene Glycol Concentration on the Synchronization of Somatic Embryos

Addition of PEG during maturation culture can effectively improve the ability of somatic embryos to mature. With the increase in the PEG concentration, the number of mature cotyledonary embryos first increased and then decreased (Figure 6B); production peaked at 80 g/L; and there was a significant decrease in the mature cotyledonary embryo numbers when the concentration of PEG was increased to 100 and 120 g/L, but there was no significant difference between them.

### Microscopy of Somatic Embryogenesis

**FIGURE 7:**
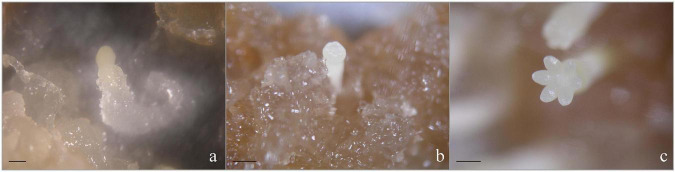
Morphology of somatic embryos at different developmental stages in *P. elliottii* × *P. caribaea.* ESMs after culturing on the solid mature medium for 14 **(a)**, 21 **(b)**, and 35 **(c)** days. Scale bar = 1 mm.

The EC maintained an efficient proliferation and potential somatic embryogenesis ability under certain PGRs in the suspension culture. In the early stage of differentiation cultured, the embryo-suspensor gathered at one end of the densely cytoplasmic cells, and the cells of the embryo head became more denser than that in the proliferation stage, forming a typical ESM, which showed significant polarity development (Figure 3b) and proliferated in a way similar to cleavage polyembryony (Hakman and Arnold, 1998). On the contrary, in the late stage of differentiation cultured, excessive cleavage polyembryony hindered the development of embryogenesis toward somatic embryos. To prevent the formation of cleavage polyembryony and promote the development of culture toward somatic embryo maturation, we added exogenous ABA and treated with low temperature accompanying the decrease of plant growth regulators, and changed the concentration and form of nitrogen. Therefore, the EC treated by the above method were highly synchronous, i.e., nearly all EC were at the stage of ESM (Figure 3c). After ESM was transferred to the mature medium, exogenous ABA accompanied with GA was added which had a very significant effect on the continuous development of ESM. In addition, the development of somatic embryos simulated the development of zygote embryos under the condition of human control. One of the important characteristics of zygotic embryo at the mature stage is that requiring a high osmotic pressure. To create a high osmotic pressure microenvironment *in vitro*, PEG was added. Polarity formed on mature medium, to the distal end to form cotyledonary primordia, and to the proximal end to form the radicle, thus forming a complete somatic embryo (Figures 7a–c). Our results showed that in the process of suspension culture, on the basis of the P6 medium, adjusting the concentration of NH_4_^+^ and NO_3_^–^ to 10 mM/L and 30 mM/L, respectively, adding 37.83 μM/L ABA at the same time, with treatment at 4°C for 12 h, and adding 80 g/L PEG to the mature medium had the best effect, i.e., more than 270 mature cotyledonary embryos were obtained per dish.

### Germination and Plant Conversion

**FIGURE 8:**
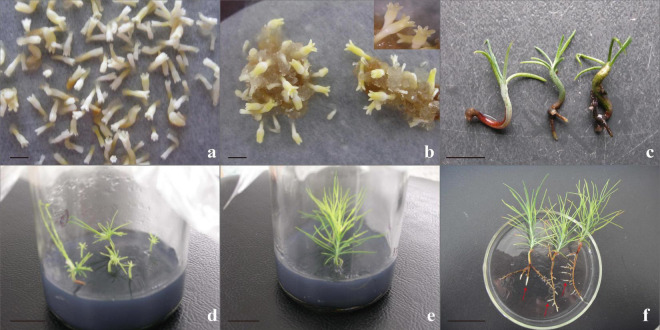
Somatic embryo germinated and conversed to form complete plants. **(a,b)** Mature somatic embryos cultured for 6–7 weeks; normal, mature cotyledonary embryos were characterized by plump cotyledons and a color of milky white or light yellow; and the number of mature cotyledonary embryos in physiology and morphology accounted for 81.2%; the statistical method was based on the characteristic parameters proposed by Le et al. (2021). **(c)** Somatic embryo germination for 20 days; the radicle turned red and become the taproot, the cotyledon turned green, hypocotyls elongated. **(d)** Somatic embryo plantlet on rooting medium for 30 days. **(e)** Intact somatic embryo-derived plant 4–5 cm in height with 2–3 roots when subcultured for 3–4 cycles. **(f)** New roots (red arrowheads) grew when cultured for 3 days in the nutrient solution. Scale bar = 1 cm.

Transferring mature cotyledonary embryos to the regeneration medium in time was essential to increasing the plantlet regeneration rate. Mature cotyledonary embryos with the character of a plump cotyledonary, elongated hypocotyl, and milky white or light-yellow color were transferred from the mature medium to the germination medium (Figures 8a,b). Cultured for 7–10 days, the radicle turned red and the hypocotyl began to elongate, and then, the radicle extended downward to form the main root (Figure 8c), about 87.5% cotyledonary embryos germinated. One month later, the germinated somatic embryos drew out needle leaves (Figure 8d) and eventually grew into a complete plantlet (Figure 8e). When the plantlet was cultured in the nutrient solution, new roots grew out quickly (Figure 8f).

## Discussion

### Establishment of a Suspension Embryogenic Cell Line

A suitable subculture transfer interval is the premise of continuous and stable proliferation and long-term embryogenic maintenance (Song et al., 2018). Shorter transfer interval led to a low growth rate that could not achieve the purpose of rapid proliferation. However, longer transfer interval resulted in a decline of proliferation multiple and browning of EC, which did not contribute to the maintenance ability of somatic embryogenesis. The growth of EC in liquid culture followed a typical S-shaped curve, but the transfer interval varied depending on the species (Fang et al., 2005; Shi et al., 2014; Wang et al., 2019). The growth of EC in *Larix* entered the logarithmic phase after 5–10 days of subculture, and the subculture period was 15 days, while in *Liriodendron* hybrids, it was 2–4 weeks (Chen et al., 2007; Song et al., 2018). In our study, the growth of EC was relatively slow within 3 days after inoculation, followed by a logarithmic phase and a stationary stage. During the logarithmic growth phase, the proliferation multiple of fresh weight and dry weight reached the highest, i.e., 7.16 and 5.32, respectively, which may be related to the rapid decline in the pH (from 5.8 to 4.2), programmed cell death (PCD), and other physiological and biochemical changes. This conclusion has also been confirmed in somatic embryogenesis of *Larix leptolepis*, that is, with a decrease of exogenous auxin content, the content of intracellular hydrogen peroxide (H_2_O_2_) reached the maximum and then promoted the occurrence of PCD (Zhang et al., 2013). Therefore, it was better to refresh the medium of hybrid *Pinus* at an interval of 9–10 days.

In the suspension culture, ESM dispersed evenly in the medium, which reduced the contact inhibition between cells and facilitated the material exchange inside and outside of the cell. Previous results showed that initial cell density had a significant effect on the proliferation and growth parameters (Shi et al., 2014; Song et al., 2018; Wang et al., 2019). In our study, we found that the proliferation multiple of ESMs cultured in liquid medium raised with increasing initial cell density. The lower initial cell densities were not beneficial to cell division; with higher amounts of initial cell densities, the cells competed for nutrients in the limited space with the increase over time, causing contact inhibition, and thus limiting the proliferation of ESM. On the contrary, due to less growth stimulus from the culture medium, the inner cells of agglomerated ESM might be restricted by the outer layers (Wang et al., 2019). The best initial density was 5 ml PCV per dish, that is to say, 2.5 (±0.1) g callus in fresh weight could be inoculated in a 50-ml liquid medium to achieve rapid proliferation of EC. The initial density was lower than that of *Pinus nigra* and *Larix* (Salaj et al., 2007; Song et al., 2018) but higher than that of *Pinus massoniana* (Shi et al., 2014), which may be related to the critical concentration of the embryogenic callus of different tree species.

### Exogenous Abscisic Acid Was Critical for the Synchronous Development of ESMs

Exogenous abscisic acid (ABA) plays an important regulatory role in the formation and development of somatic embryos in many different coniferous species (Pullman and Bucalo, 2014; Zhang et al., 2019). In this study, ESMs had different responses to exogenous ABA depending on the stage of somatic embryo development; in the early stage, ESMs were sensitive to ABA, which can reduce secondary embryogenesis and promote further development, which was consistent with the reports on *P. abies* and *L. leptolepis* (Filonova et al., 2000; Zhang et al., 2013, 2014). The fact that exogenous ABA promoted the development of ESM was probably because of the expression of target genes regulated by exogenous ABA. Based on qRT-PCR and expression analysis, ABA was the main factor in *LaGH3* and *LaTCTP* downregulation during the early stage of somatic embryogenesis, although the absence of auxin slightly decreased the expression of *LaGH3* and *LaTCTP* (Zhang et al., 2013, 2014, 2019). However, when the concentration of ABA was 18.92 μM/L, ESMs turned brown, which eliminated or decreased the potential ability of somatic embryogenesis; eventually, only a few abnormal somatic embryos are formed, and they did not have the ability to germinate, similar to what had been found with Japanese larch (*L. leptolepis*) (Zhang et al., 2013). The process of somatic embryogenesis is similar to the development of zygotic embryos (Fischerova et al., 2008; Kanno et al., 2010), which is related to PCD (Vuosku et al., 2015). Appropriate ABA concentration was necessary to promote the further development of ESM. For example, in *Picea rubens*, a relatively higher level of exogenous ABA (40 μM/L) was required to maintain normal development and inhibit the early germination of somatic embryos (Harry and Thorpe, 1991). However, in *Picea mariana* and *Picea glauca*, a lower level of ABA (12 μM/L) was suitable for embryo growth (Attree et al., 1990), and in slash pine (*P. elliottii* Engelm), the production of somatic embryos was significantly increased by adding 5 mg/L ABA during the suspension culture (Yang et al., 2020). In this study, 37.84 μM/L of ABA in the suspension culture could achieve a good synchronization effect.

### N Source Promoted the Synchronous Development of ESMs

The total nitrogen content, the nitrate/ammonium ratio, inorganic/organic nitrogen ratio, and other nitrogen sources, such as CH and amino acids, played an important role in the success of somatic embryogenesis (Zayed et al., 2012; Pìnèík et al., 2015; Wang et al., 2019). In our study, the number of embryos per dish varied between different N forms and concentrations. When the total nitrogen in the medium between 15 and 50 mM/L increased, the number of embryos per dish also increased. Nitrogen at a concentration of 40 mM/L (NH_4_^+^ 10 mM/L and NO_3_^–^ 30 mM/L or NH_4_^+^ 20 mM/L and NO_3_^–^ 20 mM/L) had a strong promoting effect on somatic embryogenesis of *P. elliottii* × *P. caribaea*. In *Cyclamen persicum* Mill, the highest number of somatic embryos was observed at 10 mM/L NH_4_^+^ and 20 mM/L NO_3_^–^ in anther cultures while at 20 mM/L NH_4_^+^ and 10 mM/L NO_3_^–^ in ovary cultures (Kiviharju et al., 1992). Our results were similar to that of *Coffea arabica*; somatic embryos can be induced by different nitrate/ammonium ratio, but an absolute requirement of ammonium in the culture medium was essential for an optimal response to somatic embryogenesis (Fuentes-Cerda et al., 2001). On the contrary, in *Phoenix dactylifera* L., the use of ammonium as the sole nitrogen source resulted in depression in somatic embryo differentiation, whereas the use of nitrate as the sole source resulted in the production of good quality somatic embryos (Zayed et al., 2012). We have also tested that the effect of nitrate and the interaction of nitrate and ammonium on somatic embryos were significantly higher than that of ammonium. It can be concluded that, when nitrate and ammonium nitrogen exist at the same time, nitrate nitrogen has a greater effect on somatic embryogenesis. The above results may be related to the fact that the modification of nitrogen forms and concentrations was an effective approach to reduce hyperhydricity, which directly affects the number of somatic embryos (Ivanova and Van Staden, 2009; El-Dawayati and Zayed, 2017).

### The Synergistic Effect of Auxin and Low Temperature on the Synchronous Development of ESMs

2,4-Dichlorophenoxyacetic acid was beneficial to the induction of SE in *Anthurium* (Bautista et al., 2008; Wang et al., 2019). However, in our study, once ESMs were induced, their proliferation no longer requires a high concentration of 2,4-D. Therefore, the concentration of 2,4-D, as well as BA and KT, should be reduced in time; otherwise, the ESM would develop toward callus. Low temperature also plays an important role in synchronization, which may be related to some reactions in the prophase of cell division. Cells that are backward in division preparation could catch up with other cells after temperature stress treatment; only a few cells could be synchronized by one treatment. If the cells were given several series of high or low temperature and were then transferred to the optimal temperature for a certain time interval, the cells could accumulate in the pre-division stage again and again, so that the vast majority of cells could be synchronized (Mei et al., 2001). In this study, the concentration of 2,4-D accompanied with BA and KT was gradually reduced. Meanwhile, the low-temperature (4°C) treatment for 12 h in each exponential growth stage obtained a better synchronous effect than the high-temperature treatments (10 or 20°C).

In the process of somatic embryogenesis, many unexpected factors, which have not been recognized and are difficult to control, lead to asynchronization of SE for the same genotype at different times. Irrespective of a solid culture system or suspension culture system, synchronization is always a bottleneck that needs to be broken thoroughly (Kumar and Nadgauda, 2014). The regulation of gene expression and the influence of the culture microenvironment in embryogenic callus culture are of great importance. We believe that the loss of embryogenesis under long-term culture was probably due to the missing key time points in the process of somatic embryogenesis and the influence of microenvironmental factors on gene expression (Lin et al., 2021), resulting in disordered gene expression, which affected the normal process of somatic embryogenesis.

With the protocol we described, it is possible to obtain a high synchronous development of somatic embryos in *P. elliottii* × *P. caribaea*; however, the mechanism of somatic embryogenesis and regulation needs further study. It has been reported that there is a very close relationship between the type and content of metal ions and the expressed proteins during the development of zygotic embryos (Zhen et al., 2008; Vuosku et al., 2015; Heringer et al., 2018). We should find suitable means and methods to simulate or create the “microenvironment” of zygotic embryo development and to ensure proper embryogenesis transition from the “Kingdom of necessity” to the “Kingdom of freedom.” Especially with the development of molecular biology and plant stem cell biology, the cloning of specific genes and the construction of cDNA libraries of genes related to embryogenesis will contribute to the exploration of the mechanism of somatic embryogenesis.

## Data Availability Statement

The original contributions presented in the study are included in the article/supplementary material, further inquiries can be directed to the corresponding author.

## Author Contributions

JC and JS contributed to the conception and design of the study. FL and JY performed the experiments. LH carried out the statistical analysis. FL wrote the manuscript. All authors contributed to manuscript revision and have read and approved the submitted version.

## Conflict of Interest

The authors declare that the research was conducted in the absence of any commercial or financial relationships that could be construed as a potential conflict of interest.

## Publisher’s Note

All claims expressed in this article are solely those of the authors and do not necessarily represent those of their affiliated organizations, or those of the publisher, the editors and the reviewers. Any product that may be evaluated in this article, or claim that may be made by its manufacturer, is not guaranteed or endorsed by the publisher.
